# The potential use of single-particle electron microscopy as a tool for structure-based inhibitor design

**DOI:** 10.1107/S2059798317004077

**Published:** 2017-04-20

**Authors:** S. Rawson, M. J. McPhillie, R. M. Johnson, C. W. G. Fishwick, S. P. Muench

**Affiliations:** a School of Biomedical Sciences and Astbury Centre for Structural Molecular Biology, University of Leeds, Leeds LS2 9JT, England; b School of Chemistry and Astbury Centre for Structural Molecular Biology, University of Leeds, Leeds LS2 9JT, England

**Keywords:** electron microscopy, structure-based drug design, conformational dynamics, membrane proteins

## Abstract

Recent developments in electron microscopy (EM) have led to a step change in our ability to solve the structures of previously intractable systems, especially membrane proteins and large protein complexes. This has provided new opportunities in the field of structure-based drug design, with a number of high-profile publications resolving the binding sites of small molecules and peptide inhibitors. There are a number of advantages of EM over the more traditional X-ray crystallographic approach, such as resolving different conformational states and permitting the dynamics of a system to be better resolved when not constrained by a crystal lattice. There are still significant challenges to be overcome using an EM approach, not least the speed of structure determination, difficulties with low-occupancy ligands and the modest resolution that is available. However, with the anticipated developments in the field of EM, the potential of EM to become a key tool for structure-based drug design, often complementing X-ray and NMR studies, seems promising.

## Abstract 2

Recent developments in electron microscopy have provided new opportunities in the field of structure-based drug design. This review looks at the challenges that remain and the future prospects for the use of electron microscopy in this area.

## 1. Introduction   

The world is in constant need of new therapeutics to treat a range of pathogens and disorders such as infectious diseases, cancer and Alzheimer’s. However, the process of drug discovery can be slow and fraught with numerous hurdles and unforeseen difficulties. From the discovery of the initial ‘hit’ compound, the time taken for a compound to reach the clinic is over ten years, owing to the processes involved in clinical testing and target validation. This highlights the enormous amount of compound development, staff and research hours dedicated to turning the initial hit compound into a drug candidate. Structural information can provide two important strands of information: the first is target validation and the second is in discovering new lead compounds and improving selectivity. Common approaches to drug discovery include high-throughput screening (HTS), structure-based design (SBD) and fragment-based drug design (FBDD).

HTS is an automated process that allows large multimillion-compound libraries to be screened against a biological target. It has been the mainstay for lead identification in the pharmaceutical industry for the past two decades (Pereira & Williams, 2007 ▸; Macarron *et al.*, 2011 ▸). Typically, 10 000 compounds are screened per day to identify hits that show a therapeutic response, with hit compounds being progressed into hit-to-lead development. Structure–activity relationships (SAR) can then lead to improved potency and selectivity of the compound series by developing a balanced profile of physicochemical properties. Despite the low probability of identifying a hit, this approach has been successful in numerous drug-discovery programs (Macarron *et al.*, 2011 ▸), including those for the antiretroviral inhibitor maraviroc and the protease inhibitor tipranavir, which are used to treat HIV infection, and the antihyperglycaemia inhibitor sitagliptin, which is used to treat type 2 diabetes.

However, there are limitations to the efficacy and effectiveness of HTS (Paul *et al.*, 2010 ▸; Bakken *et al.*, 2012 ▸), not least the large chemical libraries needed (>1 million compounds), which can represent a challenge to academic research groups or small biotech companies. Moreover, the maintenance and quality control of these libraries requires sufficient time and resources. Library compounds may also have poor physicochemical properties, such as low solubility, or functional groups associated with ‘frequent hitter’ behaviour, which results in a high percentage of false-positive results (McGovern *et al.*, 2002 ▸; Irwin *et al.*, 2015 ▸). These large libraries cover only a small fraction of chemical space, which may hinder the discovery of inhibitors for targets with unusual binding sites such as allosteric sites or protein–protein interactions (PPIs). To reduce the cost of HTS, new strategies involving smaller numbers of diverse compounds are being sought (Crisman *et al.*, 2007 ▸; Zhu *et al.*, 2013 ▸; Nissink *et al.*, 2014 ▸).

Structure-based design (SBD) utilizes prior structural knowledge of the target system to design new inhibitors and can be used to complement HTS methods *via* the structural development of an HTS ‘hit’, or as an independent approach such as identifying new leads *via* molecular docking or *de novo* design. Molecular docking, or more commonly virtual high-throughput screening (vHTS), can be used to computationally screen libraries of compounds from databases, such as ZINC15 (Sterling & Irwin, 2015 ▸), against the desired target and identifies compounds which are predicted to bind with high affinity (Rognan, 2013 ▸). Structure-based design has played a pivotal role in the discovery of close to 20 drugs in clinical use (Irwin & Shoichet, 2016 ▸), with the most well documented examples including the peptidomimetic HIV protease inhibitors nelfinavir, amprenavir and lopinavir.

Fragment-based drug discovery (FBDD) combines elements of both SBD and HTS, and has gained significant momentum as a drug-discovery platform in the last 20 years (Zartler, 2014 ▸; Erlanson *et al.*, 2016 ▸). Fragments are of lower molecular weight (<300 Da) and lipophilicity (<3) than drug-like molecules and are usually defined as having less than 20 heavy atoms. Fragment libraries consist of a few thousand molecules, thus being several orders of magnitude smaller than HTS collections, and the sampling of chemical space is more efficient (Erlanson *et al.*, 2016 ▸). Two FDA-approved drugs, the BRAF kinase inhibitor vemurafenib (Bollag *et al.*, 2012 ▸) and the BCL-2 inhibitor venetoclax (Souers *et al.*, 2013 ▸), are examples of oncology drugs discovered using FBDD.

While X-ray crystallography has provided a wealth of structural information for structure-based discovery pipelines, there are a number of limitations. These include difficulties in obtaining high-quality crystals of the protein of interest (Niedzialkowska *et al.*, 2016 ▸), crystal-packing artefacts in the structure and trapping only a static snapshot of the protein–inhibitor complex (Steuber *et al.*, 2006 ▸; Davis *et al.*, 2008 ▸). These problems, in particular the generation of high-quality crystals, have a significant impact in the membrane-protein field. For example, despite the PDB containing over 100 000 deposited X-ray crystal structures of proteins, only a small number (∼3600 as of October 2016) are membrane proteins, illustrating the difficulty in obtaining crystals of this important class of proteins. Although new approaches have been developed to improve this, with some success in the use of crystallization techniques such as lipidic cubic phase, it still proves a major challenge to reliably crystallize numerous membrane-protein families (Caffrey, 2015 ▸). This lack of structural information in turn limits the use of structure-based drug design against a number of important membrane proteins and thus hinders the development of potential therapeutics. This is not just the case for membrane proteins; other potential drug targets such as viruses and large protein complexes have also proven to be difficult to crystallize for a variety of reasons, including flexibility and size. When a structure is obtained from crystallography there is also the possibility that some of the observed interactions do not fully reflect the native state, for example crystal-packing artefacts (Davis *et al.*, 2003 ▸). A further drawback in using crystal structures for structure-based drug design is that they may only reflect a handful of conformational states and not show the full dynamic range of the system.

## 2. Advantages of electron microscopy   

**Figure 1:**
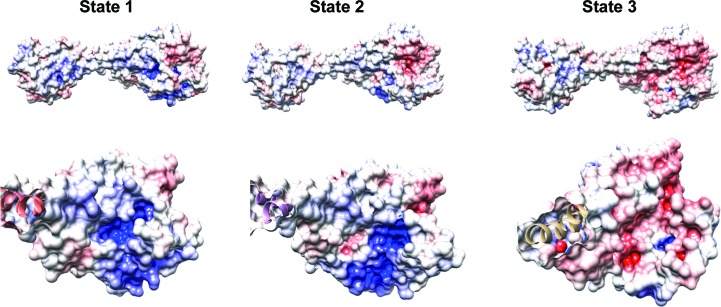
The changing shape of a potential binding pocket in V-ATPase subunit C. Atomic surface representation of yeast V-ATPase subunit C in three states showing binding-site plasticity as the complex proceeds through its catalytic cycle (PDB entries 3j9t, 3j9u and 3j9v; Zhao *et al.*, 2015 ▸). The surface is coloured by electrostatic potential, with red and blue representing negative and positive charge, respectively, showing a large positively charged cavity being exposed in state 2. This cavity is not seen within the other states of the V-ATPase complex and may provide an opportunity to lock the catalytic cycle within the second state.

Electron microscopy (EM) overcomes many of the hurdles and limitations experienced by crystallography. The first advantage is that a typical single-particle EM experiment will require microgram quantities of pure, homogenous protein, rather than the milligram quantities often required to screen for optimum crystallization conditions. The greatest advantage of EM over X-ray crystallography is it negates the need for well diffracting protein crystals, which can often create the greatest bottleneck in the crystallography pipeline. This opens up new avenues for the structural determination of previously intractable targets, from large macromolecules such as viruses to membrane proteins, where the presence of detergent makes crystallization a challenge (Bill *et al.*, 2011 ▸). EM also offers potential advantages for drug discovery by being able to trap different conformational states of a protein, moving away from static crystal structures to a dynamic ensemble of different states (Dashti *et al.*, 2014 ▸; Frank & Ourmazd, 2016 ▸). This could prove to be extremely powerful, as inhibitor-binding pockets can display plasticity and change shape as the protein samples different conformations. This dynamic range is illustrated in EM studies on the vacuolar ATPase (V-ATPase), which has been captured in three different conformational states (Zhao *et al.*, 2015 ▸). These states display significant differences, with many subunits undergoing conformational changes, for example during cycling between states subunit C changes shape and exposes a charged surface cleft (Fig. 1 ▸). This information is not seen in current crystallographic studies of the V-ATPase for a variety of reasons. Firstly, the full complex is very large (∼1 MDa) and flexible, which has made efforts to crystallize the full complex impossible thus far. Secondly, in the absence of the full complex and the interactions that it makes with other subunits it is unlikely that the isolated C subunit would adopt the three different states seen in EM, with only one state currently solved by X-ray crystallography.

**Figure 2:**
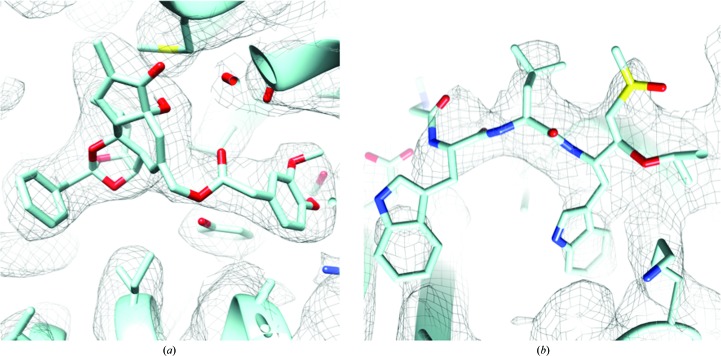
Example EM density showing bound ligands. (*a*) Resiniferatoxin ligand density from TRPV1 at 2.95 Å resolution (EMDB entry 8117, PDB entry 5irx; Gao *et al.*, 2016 ▸). (*b*) 3.6 Å resolution proteasome EM density showing bound inhibitor (EMBD entry 3231, PDB entry 5fmg; Li *et al.*, 2016 ▸). For both panels the EM density map is shown in mesh format and side chains and bound inhibitor are shown in stick format and are coloured light blue, dark blue, red and yellow for carbon, nitrogen, oxygen and sulfur, respectively.

**Figure 3:**
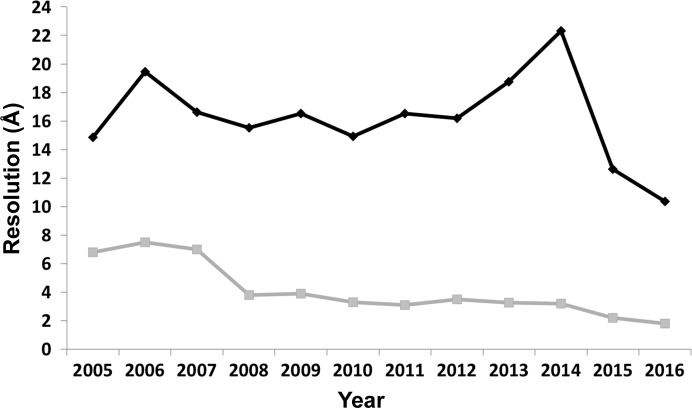
Single-particle EM resolution trends from 2005 to 2016: the change in the average (black) and highest (grey) resolution of structures determined by single-particle EM deposited in the EMDB from 2005 to 2016.

However, in the past EM has not been a viable technique for structure-based drug design, primarily owing to the low resolutions that were generally obtainable, leading to EM being known as ‘blobology’. One methodology to identify inhibitor-binding sites is through the use of tagging, which significantly increases the mass of the inhibitor, making it visible at nanometre resolution, but is limited to amenable systems (Muench *et al.*, 2014 ▸). While in the past EM has achieved approximately nanometre resolution at best for most samples, recent technological advances have now enabled near-atomic resolutions to become obtainable (Fig. 2 ▸). Improvements in microscope stability, and particularly the development of direct electron detectors, have formed the basis for the resurgence of EM as a mainstream structural technique (Smith & Rubinstein, 2014 ▸; Glaeser, 2016 ▸; Nogales, 2016 ▸). Direct electron detectors have not only increased the sensitivity and contrast of the images, but also allow the capture of high-frame-rate movies. These movies, in conjunction with new processing algorithms, allow the motion of the specimen during the exposure owing to both mechanical and beam-induced movements to be accounted for, reducing the blurring of the image and allowing higher resolution information to be recovered. Moreover, the ability to remove the later frames permits greater electron-dose exposures to be conducted, which significantly improves the contrast. Those frames which relate to the higher radiation dose can be removed, which can mitigate one of the largest remaining challenges in EM: radiation damage. With these improvements allowing higher resolution structures to be obtained, there have already been a number of structures published in complex with ligands and inhibitors (Fig. 3 ▸), from large soluble complexes such as the proteasome and β-galactosidase to smaller membrane proteins including TRP channels and gamma secretase (Bartesaghi *et al.*, 2014 ▸; Bai *et al.*, 2015 ▸; Paulsen *et al.*, 2015 ▸; Gao *et al.*, 2016 ▸; Li *et al.*, 2016 ▸).

## 3. Challenges faced by electron microscopy   

While EM offers many exciting new possibilities to play a role in, and enhance, structure-based drug design, there are still several significant challenges that hinder its widespread use. Foremost amongst these obstacles is the limited resolution that is currently obtainable. Despite the recent advances detailed in this article, EM still lags behind X-ray crystallo­graphy in that obtaining a sub-2.5 Å resolution for biological specimens is far from routine. This is a severe limitation, as details of the binding mode and precise interactions between side chains, waters and the ligand are only observable at higher resolutions. It is well established that EM can provide atomic resolution structures in the materials field, and it is routinely used for this. As such, the main factors hindering resolution in biological EM are twofold: the radiation damage sustained by the sample and the movement of the specimen itself in the electron beam.

While X-ray crystallographic studies are also affected by radiation damage, the problem is significantly worse for EM; indeed, it has been compared with a nuclear explosion at the specimen scale (Glaeser & Taylor, 1978 ▸; Orlova & Saibil, 2011 ▸). As direct detectors allow the capture of movie frames, this allows the use of all of the data, including later frames from the exposure, to align the images, giving higher contrast and easier alignment. A subset of these frames with lower dose is then used to perform the reconstructions, reducing the effects of radiation damage. However, it has been estimated that a significant loss of high-resolution information occurs after doses of around 3 e^−^ Å^−2^ (Baker *et al.*, 2010 ▸; Vinothkumar *et al.*, 2014 ▸; Grant & Grigorieff, 2015 ▸), and much of this dose occurs within the first moments of the exposure. Moreover, the frames which would contain this high-resolution information suffer from the worst effects of beam-induced motion, whereby movements occur as the grid is first exposed to the electron beam through poorly understood combinations of charging and expansion/contraction of the vitreous ice and the support film, which is typically made of amorphous carbon (Brilot *et al.*, 2012 ▸; Glaser *et al.* 2011 ▸). This means that with current methodology these frames cannot be used, making extremely high resolution (∼1 Å) structures unlikely in the near future, although research into new support substrates which may limit the problems associated with grid expansion and improve stability is ongoing (Russo & Passmore, 2014 ▸, 2016 ▸). Radiation damage can also have a more subtle effect aside from the degradation of resolution, where damage causes protein side chains to shift, which could lead to subtle differences in the inhibitor binding position. Previous studies have shown that negative side chains appear to suffer radiation damage preferentially (Allegretti *et al.*, 2014 ▸; Bartesaghi *et al.*, 2014 ▸; Grant & Grigorieff, 2015 ▸), so if there are key negatively charged residues in the binding pocket then this may induce a significant movement of the inhibitor or show a binding mode which is not physiologically relevant. However, it should be noted that weak density for negative side chains can also be a feature of the electric potential map generated in the electron-microscopy experiment (Wang & Moore, 2017 ▸) and is not in all cases radiation damage.

While EM can tackle proteins that are intractable for crystal studies, such as large complexes and membrane proteins, the current limitations in technology impose a size limit on the proteins that can be observed. Currently, proteins smaller than ∼200 kDa prove a major challenge for EM, although there are exceptions to this (Merk *et al.*, 2016 ▸). While developments are under way to improve this, and while there has already been significant progress through the use of direct detectors, this size limitation still hinders the use of EM for several important classes of drug targets, including GPCRs. As such, this is the subject of much ongoing research, particularly the development of phase plates (discussed in more detail below).

A further drawback of using an EM approach for structure-based drug-design purposes is the speed at which structures can be obtained. While in crystallography it is routine to set up several co-crystallizations or soaks simultaneously and the data collection for each crystal obtained is very rapid (minutes), EM data collection is still relatively slow, typically taking several days of collection for a single sample. This is particularly an issue for fragment-based discovery: whereas crystallography programs such as XChem, developed at the Diamond Light Source (UK), are capable of screening up to 500 crystals in a single day for bound fragments, this scale of throughput is not yet possible for EM studies. The primary reason for this is the typical requirement for over 100 000 ‘particles’, which in the absence of symmetry can require in excess of 2000 micrographs. This level of data can be achieved within 2–3 days of microscope time using automatic data-collection runs on the microscope. Processing of the resulting data can also take significant time, of the order of weeks to months to extract the most from the raw data, currently precluding EM as a high-throughput structural technique. Tied to this is the volume of data that is produced in a single EM experiment, with a single micrograph being between 1 and 10 GB in size, leading to data sets of >10 TB for a single specimen. This presents a major infrastructure challenge, and makes the initial barrier to entry for EM substantial without significant resources dedicated to processing and data storage. Nevertheless, improvements are being made in the speed of data acquisition and enhancing the speed of image processing. Fragment-based screening is particularly challenging for EM owing to issues of low occupancy, as a large number of individual molecules need to be averaged to obtain high-resolution reconstructions, and thus inhibitor density will be lost with partial occupancy. This may be overcome to some extent if the binding of the fragment induces a large conformational change that could be identified through classification of the data set such that ‘bound’ and ‘unbound’ can be computationally sorted. However, unlike crystallographic studies, the ability to raise the ligand concentration significantly above the *K*_d_, for example through crystal soaking, becomes challenging, especially for peptide inhibitors, where a large excess will result in significant reductions in contrast within the cryo-EM image.

## 4. Future potential   

EM has a promising role to play in structure-based drug-design approaches, despite the current challenges that need to be addressed. One of the key limitations that EM must overcome in order to hold widespread appeal in drug development is the size limitation of the proteins that can be studied. This limitation is based around the low signal-to-noise ratio (SNR) and the low contrast of biological samples in EM at typical dose ranges (∼20 e^−^ Å^−2^). The increased SNR from the advent of direct detectors has already allowed a decrease in the minimum molecular weight from ∼500 to ∼150–200 kDa. Further increases in contrast and the SNR can be gained through phase-plate technology, which is under development by several groups and companies, with the Volta phase plate already allowing the structures of human Prx3 (∼250 kDa) and the nucleosome core particle to be determined (Chua *et al.*, 2016 ▸; Khoshouei *et al.*, 2016 ▸). Both phase plates and direct detectors are still in their infancy; with further development and optimization this technology could enable EM to look at a much wider range of proteins than are currently viable.

Another method in development to increase the range of viable proteins for study *via* EM is through the use of affinity grids. Structural studies of several potentially therapeutically interesting proteins are precluded simply by difficulties in obtaining sufficiently pure protein to carry out EM studies. This can be owing to challenges in generating sufficient yields because of difficulty in overexpressing functional protein owing to factors such as cell toxicity, poor folding, recycling and expense in large-volume expression, especially with HEK cells. To combat this, rather than performing traditional large-scale overexpression and purification and studying the resulting protein *via* EM, work is ongoing to purify the sample directly onto the EM grid (Benjamin *et al.*, 2016 ▸; Yu *et al.*, 2016 ▸). Various methods are being developed to achieve this, from immobilizing antibodies to the grid surface to tethering the protein of interest to the grid (requiring no prior genetic manipulation) or alternatively applying nickel-doped lipid monolayers to the grid surface to extract His-tagged protein directly. While currently the only examples of this work at high resolution have been highly symmetrical virus structures, with further development it could become a powerful technique for the structural study of hard-to-obtain proteins (Yu *et al.*, 2016 ▸).

General advances in microscopes, including the use of spherical aberration (*C*_s_) correction, which has already been shown to aid in obtaining high-resolution structural information (Fischer *et al.*, 2015 ▸), and detectors should enable further increases in resolution, allowing more valuable information about ligand binding to be extracted from the resulting reconstructions. In addition to this, computational power is constantly increasing, meaning that the computational bottlenecks that currently hinder the rapid processing of EM data should lessen over time. At the same time, graphical acceleration of the processing pipeline using GPU technology is an active area of research, with several key steps already having been optimized in this manner, cutting processing times for these steps from days to hours (Zhang, 2016 ▸). Further work is ongoing in utilizing this dramatic acceleration to speed up the currently slow steps of classification and refinement. With the rapid rate of change in computational power and developments such as GPU acceleration, it is easy to envisage a processing pipeline for EM where several processing steps are carried out ‘on the fly’ as data are collected, in an analogous manner to X-ray data collection at many synchrotron sources (Lander *et al.*, 2009 ▸; la Rosa-Trevín *et al.*, 2016 ▸).

Advances in technology and computation may also allow EM to study biological processes in a time-dependent manner, giving insight into the dynamic changes that occur within the system. Work is ongoing to develop time-resolved EM through a variety of methods, including rapid mixing/spraying in order to physically trap the protein of interest in a variety of functional states (Walker *et al.*, 1995 ▸; Lu *et al.*, 2009 ▸; Chen & Frank, 2016 ▸), as well as computational manifold mapping approaches, which have already been utilized to identify several functional conformational states of the ribosome (Dashti *et al.*, 2014 ▸; Frank & Ourmazd, 2016 ▸). By removing the constraints of a crystal lattice, it is possible to map the larger conformational changes associated with catalytic cycling, which is invaluable in understanding the full dynamic range of a protein–protein complex. Only by moving away from ‘static’ snapshots can we fully understand the plasticity of inhibitor-binding pockets and identify new binding sites.

For membrane proteins, a significant challenge, especially for structure-based inhibitor design, is to study the protein in a more native state that better reflects the target structure. New methodologies for extracting membrane proteins in more native states have been reported, for example styrene maleic acid lipid particles (SMALPs), which extract membrane proteins with their native lipids rather than in detergent micelles, and have been used for EM analysis (Postis *et al.*, 2015 ▸; Lee *et al.*, 2016 ▸). Furthermore, saposonin–lipoprotein nanoparticles have shown promise in high-resolution cryo-EM studies on membrane proteins (Frauenfeld *et al.*, 2016 ▸). Nanodiscs also offer the potential to create more native environments that can be adapted for different lipid environments (Gao *et al.*, 2016 ▸). However, there is a limitation with all of these technologies in that they all represent an ‘open’ system whereby a chemical gradient or membrane potential cannot be generated. This is particularly pertinent for ion channels and transporters, where the native conformational state cannot be seen in the absence of such a gradient. This provides a challenge for structure-guided inhibitor design where the structure of the conformational state which predominates in the natural system is not seen. To overcome these challenges it is beneficial to reconstitute the membrane protein within a proteoliposome, which can maintain such a gradient. The use of this approach has been used to investigate the BK channel and mouse serotonin 5-HT_3_ (Wang & Sigworth, 2009 ▸; Kudryashev *et al.*, 2016 ▸), and with improved data-collection speeds and processing approaches this could become a powerful means of studying membrane proteins in more ‘native’ environments, better reflecting the different conformational states that are accessible.

## 5. Concluding remarks   

Although the use of EM as a tool for structure-based drug design is in its infancy and has not yet been reported to actively design new lead compounds, it is clear that it offers a wide range of new opportunities. While the resolution of EM is constantly increasing and new technological developments will only accelerate this, it is likely that X-ray crystallography will remain the gold-standard technique for atomic resolution information in the near future. However, EM can provide a wealth of potentially valuable complementary information, including insight into conformational dynamics and, crucially, high-resolution structural information from a more native state freed from the constraints of the crystal lattice. This could provide a vital contribution to the drug-design pipeline through both target-validation and structure-guided approaches.
